# Cultivation-Independent Screening Revealed Hot Spots of IncP-1, IncP-7 and IncP-9 Plasmid Occurrence in Different Environmental Habitats

**DOI:** 10.1371/journal.pone.0089922

**Published:** 2014-02-24

**Authors:** Simone Dealtry, Guo-Chun Ding, Viola Weichelt, Vincent Dunon, Andreas Schlüter, María Carla Martini, María Florencia Del Papa, Antonio Lagares, Gregory Charles Auton Amos, Elizabeth Margaret Helen Wellington, William Hugo Gaze, Detmer Sipkema, Sara Sjöling, Dirk Springael, Holger Heuer, Jan Dirk van Elsas, Christopher Thomas, Kornelia Smalla

**Affiliations:** 1 Julius Kühn-Institut – Federal Research Centre for Cultivated Plants (JKI), Institute for Epidemiology and Pathogen Diagnostics, Braunschweig, Germany; 2 Division of Soil and Water Management, KU Leuven, Heverlee, Belgium; 3 Center for Biotechnology (CeBiTec), Institute for Genome Research and Systems Biology, Bielefeld University, Bielefeld, Germany; 4 IBBM (Instituto de Biotecnología y Biología Molecular), CCT-CONICET-La Plata, Departamento de Ciencias Biológicas, Facultad de Ciencias Exactas, Universidad Nacional de La Plata, La Plata, Argentina; 5 School of Life Sciences, University of Warwick, Warwick, United Kingdom; 6 Laboratory of Microbiology, Wageningen University, Wageningen, The Netherlands; 7 Södertörns högskola (Sodertorn University), Inst. för Naturvetenskap, Miljö och medieteknik (School of Natural Sciences, Environmental Studies and media tech), Huddinge, Sweden; 8 University of Groningen, Groningen, The Netherlands; 9 School of Biosciences, University of Birmingham, Edgbaston, Birmingham, Warwick, United Kingdom; Institut National de la Recherche Agronomique, France

## Abstract

IncP-1, IncP-7 and IncP-9 plasmids often carry genes encoding enzymes involved in the degradation of man-made and natural contaminants, thus contributing to bacterial survival in polluted environments. However, the lack of suitable molecular tools often limits the detection of these plasmids in the environment. In this study, PCR followed by Southern blot hybridization detected the presence of plasmid-specific sequences in total community (TC-) DNA or fosmid DNA from samples originating from different environments and geographic regions. A novel primer system targeting IncP-9 plasmids was developed and applied along with established primers for IncP-1 and IncP-7. Screening TC-DNA from biopurification systems (BPS) which are used on farms for the purification of pesticide-contaminated water revealed high abundances of IncP-1 plasmids belonging to different subgroups as well as IncP-7 and IncP-9. The novel IncP-9 primer-system targeting the *rep* gene of nine IncP-9 subgroups allowed the detection of a high diversity of IncP-9 plasmid specific sequences in environments with different sources of pollution. Thus polluted sites are “hot spots” of plasmids potentially carrying catabolic genes.

## Introduction

The search for novel enzymes able to degrade recalcitrant natural contaminants such as chitins and lignins and man-made pollutants such as halogenated aliphatic and aromatic compounds motivated metagenomic explorations of various environments. It has been observed that the microbial metagenomes of open ecosystems, including soils and aquatic habitats, clearly represent rich reservoirs of genes that determine the desired enzymatic reactions in which chitinases, ligninases and dehalogenases are involved [1], [2]. By anthropogenic activities, recalcitrant compounds have also been released as environmental pollutants. Typical metagenomic approaches employ genetic or activity screens of cloned large DNA fragments from various environments [3]. However, the idea of capturing complete mobile genetic elements (MGE) into suitable recipients might be an alternative and complementary approach to access the genes coding for novel enzymes or even complete degradative pathways. Mobile genetic elements such as plasmids are often found to play an important role in the adaptation of bacterial communities to changing and, due to pollutants, often challenging environmental conditions. For example, partial or complete degradative pathways were previously reported to be localized on plasmids belonging to the IncP-1, IncP-7 or IncP-9 group [1]. The present study aimed to monitor various environments for the abundance of these plasmids by using a cultivation-independent total community (TC-) DNA based approach to select the most promising habitats for mining plasmids potentially carrying genes coding for novel enzymes. We hypothesized that the frequency of occurrence of genes encoding the desired enzymatic activities is increased in the MGE gene pool. In particular, plasmids belonging to the incompatibility groups (Inc) P-1, P-7 and P-9 often carry genes responsible for the degradation of xenobiotic (man-made) and natural organic pollutants, being essential players in the adaptation of bacterial communities to new toxic compounds released in the environment [1]. Therefore, selected natural or treated environments were analyzed for the prevalence of plasmids belonging to the IncP-1, IncP-7 and IncP-9 groups by a cultivation-independent approach. Some of these environments were enriched for the desired degradation function by adding the relevant substrates, i.e. chitin, lignin and/or organohalogens. The habitats sampled included a variety of soils (one soil sample amended with chitin, peat bogs), biopurification systems (BPS) for pesticide removal from contaminated water, biogas production plants, wastewater, as well as aquatic (river bank sediments, sponges) environments from a wide range of geographic regions. Total community DNA was analyzed for the presence of IncP-1, IncP-7 and IncP-9 plasmids by means of PCR and subsequent Southern blot hybridization. A novel primer system for the specific amplification of IncP-9 plasmids was developed and tested in the present study. Southern blot hybridization using probes derived from reference plasmids belonging to different subgroups of IncP-1 plasmids provided new insights into their environmental dissemination. Our results showed a particularly widespread dissemination of IncP-1 plasmid-specific sequences. Different hot spots of plasmid occurrence were identified.

## Materials and Methods

### Ethics Statement of Provided Samples

None of the samples used in the present work involved any endangered or protected species. The marine sponges were obtained under legal permits from competent authorities: *Halichondria panicea* was obtained under a permit from authorities given to Wageningen University, while *Corticium candelabrum* and *Petrosia ficiformis* marine sponges were sampled under a Spanish permit to CEAB-CSIC. The sediments and soil originated from UK were taken from a river bed accessed from a public right of way and therefore no permissions were needed. Landsort Deep was sampled from a national environmental monitoring site (BY31) in conjunction with the Baltic Sea monitoring programme. The sampling permission was provided by the Stockholm University marine research Center. The Askö samples were sampled at the Stockholm Marine Research Center (now the Stockholm University Marine Research Center. The sediment and soils from Argentina were obtained from public locations as part of fundamental studies performed through a collaborative project with the agreement of the Facultad de Ciencias Exactas, Universidad Nacional de La Plata, and did not require any specific permission. The biopurification systems (BPS) samples were obtained from private land with permission from the local farmers in Kortrijk, Leefdaal, Lierde and Koksijde, located in Belgium.

### Extraction of Total Community DNA (TC-DNA) and Metagenomic DNA (Pooled Fosmid Library) from Different Environmental Samples

The TC-DNA and/or metagenomic DNA (metagenomic DNA represented by the metagenomic pooled fosmid library from Baltic Sea**)** from different environmental samples originating from various geographic regions were extracted using different methods. The protocols used for TC-DNA extraction of each sample type are given in Table 1.

**Table 1:** Description of environmental samples analyzed and TC-DNA extraction applied.

Samples	Description of samples	TC-DNA extraction method
**A**	Biogas production plant fermentation sample from Bielefeld, Germany	[24]
**B.1**	*Biopurification system (BPS) from Leefdaal, Belgium	[17]
**B.2**	BPS from Leefdaal, Belgium	[17]
**B.3**	BPS from Leefdaal, Belgium	[17]
**C.1**	*BPS from Belgium (Pcfruit)	[17]
**C.2**	BPS from Belgium (Pcfruit)	[17]
**C.3**	BPS from Belgium (Pcfruit)	[17]
**C.4**	BPS from Belgium (Pcfruit)	[17]
**C.5**	BPS from Belgium (Pcfruit)	[17]
**C.6**	BPS from Belgium (Pcfruit)	[17]
**D.1**	*BPS from Lierde, Belgium	[17]
**D.2**	BPS from Lierde, Belgium	[17]
**D.3**	BPS from Lierde, Belgium	[17]
**E.1**	*BPS from Kortrijk, Belgium	[17]
**E.2**	BPS from Kortrijk, Belgium	[17]
**E.3**	BPS from Kortrijk, Belgium	[17]
**F.1**	*BPS from Koksijde, Belgium	[17]
**F.2**	BPS from Koksijde, Belgium	[17]
**F.3**	BPS from Koksijde, Belgium	[17]
**G.1**	Soil from La Plata, Argentina polluted with industrial residues and petrol	[18]
**G.2**	Soil from La Plata, Argentina polluted with industrial residues and petrol	[18]
**G.3**	Soil from La Plata, Argentina polluted with industrial residues and petrol	[18]
**H.1**	Sediments from La Plata, Argentina polluted with pesticides and petrol	[18]
**H.2**	Bordering soil from a water channel in La Plata, Argentina polluted with pesticides, residues from paper industry	[18]
**H.3**	Bordering soil from a water channel in La Plata, Argentina polluted with pesticides, residues from paper industry	[18]
**J**	Marginal river forest soil from La Plata, Argentina polluted with industrial residues	[18]
**L.1**	Bordering soil from a water channel in Buenos Aires, Argentina polluted with industrial residues	[18]
**L.2**	Bordering soil from a water channel in Buenos Aires, Argentina polluted with industrial residues	[18]
**L.3**	Bordering soil from a water channel in Buenos Aires, Argentina polluted with industrial residues	[18]
**M**	*Halichondria panicea* (marine sponge) from Oosterschelde, Netherlands	[25]
**N**	*Corticium candelabrum* (marine sponge) from Punta Santa Anna (Blanes), Spain	[25]
**O**	*Petrosia ficiformis* (marine sponge) from Punta Santa Anna (Blanes), Spain	[25]
**P.1**	Askö sediment from Baltic Sea Sweden (bottom fraction - anoxic)	[26]
**P.2**	Askö sediment from Baltic Sea Sweden (middle fraction - mixed anoxic/oxic)	[26]
**P.3**	Askö sediment from Baltic Sea Sweden (top fraction - oxic)	[26]
**Q**	Pooled fosmid library, Askö sediment, Baltic Sea	[3]
**R**	Landsort in Sweden	[26]
**S.1**	Sediment from a river in Warwickshire, UK	[27]
**S.2**	Sediment from a river in Warwickshire, UK	[28]
**T**	Soil from UK amended with chitin (Test site 1)	[28]

*BPS samples received water contaminated with different types of pesticides from spillage and residue water collected when cleaning the spraying equipment such as ethofumesate, fenpropimorf, fluroxypyr, glyphosate, linuron, metamitron and S-metalochlor (information provided by the farmers).

### 16S rRNA Gene PCR Amplification and Quantification

16S rRNA gene PCR amplification reaction was done as previously described by Heuer *et al.* (2009) [5] (product size of 1506 bp). The quality of the PCR product was determined by electrophoresis in 1% agarose gel and visualized with ethidium bromide staining and under UV light by comparison with the 1-kb gene-rulerTM DNA ladder (Fermentas, St Leon-Rot, Germany). Quantitative PCR (qPCR) targeting the 16S rRNA gene was performed with the TaqMan system as described by Suzuki *et al.* (2000) [6]. The 16S rRNA gene qPCR standard was made from cloned 16S rRNA gene amplicons (1467 bp) of *E. coli.* and 10^9^, 10^8^, 10^7^ 16S rRNA gene copy numbers were used.

### Southern Blot-PCR Based Detection of IncP-1 Plasmids

IncP-1 plasmids belonging to the α, β, γ, δ and ε subgroups were detected based on the amplification of the *trfA* region (product size of 281 bp) from TC-DNA and metagenomic DNA using the primers described by Bahl *et al*. (2009) [2]. Digoxygenin-labeled probes targeting different IncP-1 plasmids subgroups were generated from reference plasmids belonging to the IncP-1α, β, γ, δ and ε plasmids (Table 2). The IncP-1 mixed probe was prepared by mixing probes generated for the different subgroups. The random primed digoxigenin labeling of PCR amplicons excised from preparative agarose gels was done according to the Roche manufacturer’s protocol (Roche Diagnostics Deutschland GmbH, Mannheim, Germany).

**Table 2:** Generation of probes for Southern blot hybridization.

Probe	Reference plasmid	Plasmids host strain	Primers
IncP-1α	RP4	*E. coli*	[2]
IncP-1β	R751	*E. coli CM544*	[2]
IncP-1γ	pQKH54	*E. coli DH10B*	[2]
IncP-1δ	pEST4011	*Alcaligenes xylosoxidans EST4002*	[2]
IncP-1ε	p3-408	*E. coli cv601-GFP*	[2]
IncP-7	pCAR1,	*Pseudomonas resinovorans CA10*	[4]
IncP-9 α	pM3	*Pseudomonas putida*	*This study*
IncP-9 β	pBS2	*Pseudomonas putida BS268*	*This study*
IncP-9 γ	pSN11	*Pseudomonas putida BS349*	*This study*
IncP-9 δ	pSN11	*Pseudomonas putida SN11*	*This study*
IncP-9 ε	pMG18	*Pseudomonas putida AC34*	*This study*
IncP-9 ζ	pNL60	*Pseudomonas* spp. *18d/1*	*This study*
IncP-9 η	pNL15	*E. coli C600*	*This study*
IncP-9 θ	pSVS15	*Pseudomonas fluorescens SVS15*	*This study*
IncP-9 ι	pNL22	*Pseudomonas* spp. *41a/2*	*This study*

### Southern Blot-PCR Based Detection of IncP-7

PCR amplification of the *rep* region of IncP-7 plasmids (product size of 524 bp) from TC-DNA was performed as previously described by Izmalkova *et al.* (2005) [4]. Southern blotted PCR amplicons were hybridized at medium stringency with the dig-labeled IncP-7 probe generated from the reference plasmid pCAR1 isolated from *Pseudomonas resinovorans* according to the manufacturer’s instructions (QIAGEN® Plasmid Mini Kit) (Table 2). The randomly primed digoxigenin labeling of PCR amplicons was done as described above.

### Analyzing the Diversity and Abundance of IncP-9 Plasmids by a Novel PCR System Targeting the *oriV-rep* Region

To study the abundance and diversity of IncP-9 plasmids, a novel PCR system targeting the *oriV-rep* regions was developed and applied to detect IncP-9 plasmids in TC- and metagenomic DNA from all samples analyzed (Table 1).

Multiple alignments of 28 sequences of *oriV* (EU499619-EU499641, AF078924, AB237655, AJ344068, AB257759 and AF491307) and *rep* (EU499644-EU499666, AF078924, AB237655, AJ344068, AB257760 and AF491307) were performed with Molecular Evolutionary Genetics Analysis (MEGA 4). Conserved regions of sequences belonging to nine IncP-9 subgroups [7] were used for the primer design. The selected primer system consists of 21-mer degenerate forward primer (5-GAG GGT TTG GAG ATC ATW AGA-3) and reverse primer (5-GGT CTG TAT CCA GTT RTG CTT-3). *In silico* analysis showed no mismatch for at least 12 bp at the 3′ end of each primer and 1–4 mismatches for each sequence type at the 5′ end (Fig. S1). The expected amplicon size is 610–637 bp. The primers were further tested with plasmid DNA from the reference plasmids summarized in Fig. S1. None of the plasmids belonging to other incompatibility groups was amplified while the reference plasmids were amplified. The reaction mixture (25 µl) contained 1 µl template DNA (1–5 ng), 1× Stoffel buffer (Applied Biosystems, Foster, CA), 0,2 mM dNTPs, 2,5 mM MgCl_2_, 2 µg/µl bovine serum albumin, 0.2 µM of each primer, and 2.5 U TrueStartTaq DNA polymerase (Stoffel fragment, Applied Biosystems). Denaturation was carried out at 94°C for 5 min, followed by 35 cycles of 1 min at 94°C, 1 min at 53°C (primer annealing) and 2 min at 72°C and a final extension of 10 min at 72°C.

PCR amplicons of *oriV-rep* regions of nine IncP-9 subgroups IncP-9 plasmids (Table 2) were gel-purified and digoxigenin-labeled as described above. Southern blot hybridization of *oriV-rep* amplicons from different environmental samples listed above was performed with a mixture of these probes under medium stringency following the manufacturer’s instructions (Roche Diagnostics Deutschland GmbH, Mannheim, Germany). Clone libraries were generated for these three BPS to confirm primer specificity. *oriV-rep* amplicons were gel-purified, ligated into pGEM vectors, and transformed into *E. coli* JM109 competent cells according to the instructions of the manufacturer. Clones containing the correct inserts were selected for sequencing. BLAST-N analysis was used to identify *oriV-rep* sequences of IncP-9. All sequences analyzed share high similarity with IncP-9 *oriV* or *rep* sequences in NCBI. The sequences and those of known *oriV-rep* sequences in the data base were aligned and phylogenetic tree was calculated according to the neighbor-joining method and bootstrapping analysis using MEGA 4.

### Nucleotide Sequence Accession Numbers of Cloned IncP-9 *oriV-rep* Gene Amplicons

Amplicon sequences have been submitted to NCBI SRA with IncP-9 *oriV-rep* gene amplicons under accession numbers KF706553 - KF706633.

## Results

### Determination of Bacterial 16S rRNA Gene Copies by qPCR

To estimate the bacterial density of the different environmental samples analyzed, 16S rRNA gene copies were determined by quantitative real-time PCR from the TC-DNA. Most of the samples (Table 3) showed a high abundance of bacterial populations ranging from 10^8^ to 10^9^ 16S rRNA gene copy numbers per gram of material. For a few samples significantly lower 16S rRNA gene copy numbers per gram of material (Tukey’s test p>0.05) were detected (Table 3).

**Table 3:** Bacterial densities and PCR-Southern blot hybridization detection of plasmid replicon-specific sequences belonging to the five IncP-1 subgroups, IncP-7 and IncP-9.

Sample	Description of samples	P-1	α	β	ε	γ	δ	P-7	P-9	16Slog10/g
**A**	Biogas production plant from Bielefeld, Germany	+++	++	++	++	–	++	–	–	9,34
**B.1**	Biopurification system (BPS) from Leefdaal, Belgium	+++	*–*	++	+++	++	+++	+++	+++	9,32
**B.2**	BPS from Leefdaal, Belgium	+++	–	++	+++	++	+++	+++	+++	9,25
**B.3**	BPS from Leefdaal, Belgium	+++	–	++	+++	++	++	+++	+++	8,43
**C.1**	BPS from Belgium (Pcfruit )	+++	+	+++	+++	++	++	+++	+++	9,32
**C.2**	BPS from Belgium (Pcfruit )	+++	+	+++	++	++	+++	+++	+++	8,28
**C.3**	BPS from Belgium (Pcfruit )	++	–	++	++	–	+++	++	+	8,36
**C.4**	BPS from Belgium (Pcfruit )	+	–	++	+	+	+++	+++	+++	8,54
**C.5**	BPS from Belgium (Pcfruit )	+++	(+)	+++	+++	++	+++	+++	+++	8,66
**C.6**	BPS from Belgium (Pcfruit )	+	–	++	++	+	++	++	–	8,15
**D.1**	BPS from Lierde, Belgium	+++	–	++	–	–	+++	++	++	8,61
**D.2**	BPS from Lierde, Belgium	+++	–	++	++	–	+++	++	++	8,59
**D.3**	BPS from Lierde, Belgium	+++	–	++	+++	++	+++	++	++	8,31
**E.1**	BPS from Kortrijk, Belgium	+++	–	+++	+++	+++	+	+++	++	9,2
**E.2**	BPS from Kortrijk, Belgium	+++	–	+++	+++	+++	++	+++	+++	9,03
**E.3**	BPS from Kortrijk, Belgium	+++	–	++	++	+++	–	+++	+++	9,11
**F.1**	BPS from Koksijde, Belgium	++	(+)	++	+	–	+++	(+)	+++	9,01
**F.2**	BPS from Koksijde, Belgium	++	+++	++	–	–	++	–	+++	8,9
**F.3**	BPS from Koksijde, Belgium	++	(+)	++	+	–	+++	–	+++	8,95
**G.1**	Soil from La Plata, Argentina	+++	(+)	+	+	–	+++	+++	+++	8,55
**G.2**	Soil from La Plata, Argentina	+++	–	(+)	+	++	+	+++	+++	8,53
**G.3**	Soil from La Plata, Argentina	+++	–	++	–	++	+	–	(+)	8,22
**H.1**	Sediments from La Plata, Argentina	+++	++	+++	++	++	+++	+++	+++	8,96
**H.2**	Bordering soil from a water channel in La Plata, Argentina	+++	++	++	++	++	+++	+	+++	8,49
**Sample**	**Description of samples**	**P-1**	**α**	**β**	**ε**	**γ**	**δ**	**P-7**	**P-9**	**16S** **log10/g**
**H.3**	Bordering soil from a water channel in La Plata, Argentina	+++	+	++	++	++	+++	–	+++	8,7
**I**	Sweet-water soil from a river in La Plata, Argentina	+++	+	++	++	++	++	+++	+++	7,91
**J**	Marginal river forest soil from La Plata, Argentina	++	–	–	–	–	–	–	–	8,32
**L.1**	Bordering soil from a water channel in Buenos Aires, Argentina	+++	–	++	+	–	++	–	–	8,29
**L.2**	Bordering soil from a water channel in Buenos Aires, Argentina	+++	+	+++	++	++	+++	–	(+)	8,6
**L.3**	Bordering soil from a water channel in Buenos Aires, Argentina	+++	–	++	++	++	+++	++	+++	7,66
**M**	*Halichondria panicea* (marine sponge) from Oosterschelde, Netherlands	++	–	++	–	–	+++	–	–	7,32
**N**	*Corticium candelabrum* (marine sponge) from Punta Santa Anna (Blanes), Spain	++	–	+	–	–	+	–	–	8,18
**O**	*Petrosia ficiformis* (marine sponge) from Punta SantaAnna (Blanes), Spain	++	–	++	–	–	+	–	–	8,4
**P.1**	Askö sediment from Baltic Sea Sweden (bottom fraction - anoxic)	++	–	–	–	–	+	–	–	8,34
**P.2**	Askö sediment from Baltic Sea Sweden (middle fraction mixed anoxic/oxic)	++	++	+++	++	–	+	–	–	8,43
**P.3**	Askö sediment from Baltic Sea Sweden (top fraction - oxic)	+++	–	++	+++	–	+	–	–	8,09
**Q**	Pooled fosmid library, Askö sediment, Baltic Sea	+++	++	+	+	–	+	–	–	5,01
**R**	Landsort in Sweden	+++	++	–	–	–	–	–	–	8,16
**S.1**	Sediment from a river in Warwick, UK	+++	/	/	/	/	/	–	(+)	5,78
**S.2**	Sediment from a river in Warwick, UK	+++	/	/	/	/	/	++	–	6,26
**T**	Soil from Cuba amended with chitin (Test site 1)	+++	/	/	/	/	/	+++	++	6,95
**negative control**		–	–	–	–	–	–	–	–	
**RP4 (IncP-1α)**		+++	+++							
**R751 (IncP-1β)**		+++		+++						
**pKJK5 (IncP-1ε)**		++			++					
**pQKH54 (IncP-1γ)**		+++				+++				
**pEST4011 (IncP-1δ)**		+++					+++			
**pCAR1 (IncP-7)**								+++		
**pNF 142 (IncP-9)**									+++	

Hybridization signal: (+++) very strong, with exposure time up to five minutes; (++) strong, with exposure time up to one hour; (+) weak, with exposure time up to three hours; (−) none, with exposure time of more than three hours; (/) not analyzed.

### Distribution of IncP-1 Plasmids in Different Environments

**Figure 1:**
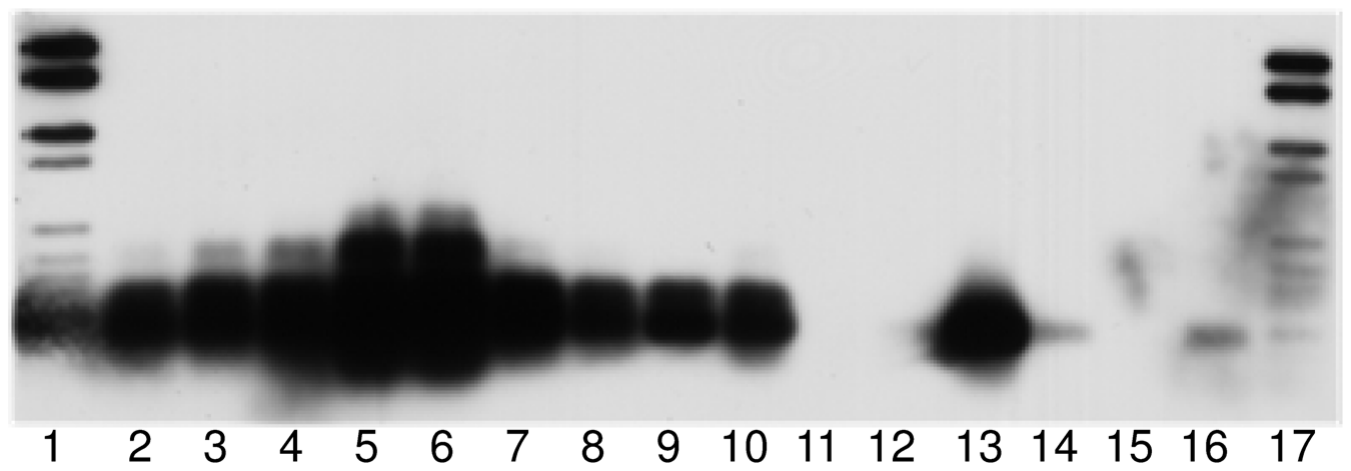
Biopurification systems (BPS). Hybridization of Southern-blotted PCR products obtained with *trfA* primer system from TC-DNA of BPS (IncP-1β specific group). Lanes: 1 and 17, dig ladder; lanes 2 to 4, BPS from Lierde, Belgium; lanes 5 to 7, BPS from Kortrijk, Belgium; lanes 8–10, BPS from Koksijde, Belgium; lane 11, negative control; lanes 12–16, IncP-1 positive controls RP4 (α), R751 (β), pKJK5 (ε), pQKH54 (γ) and pEST4011 (δ). Exposure time of 5 min.

**Figure 2:**
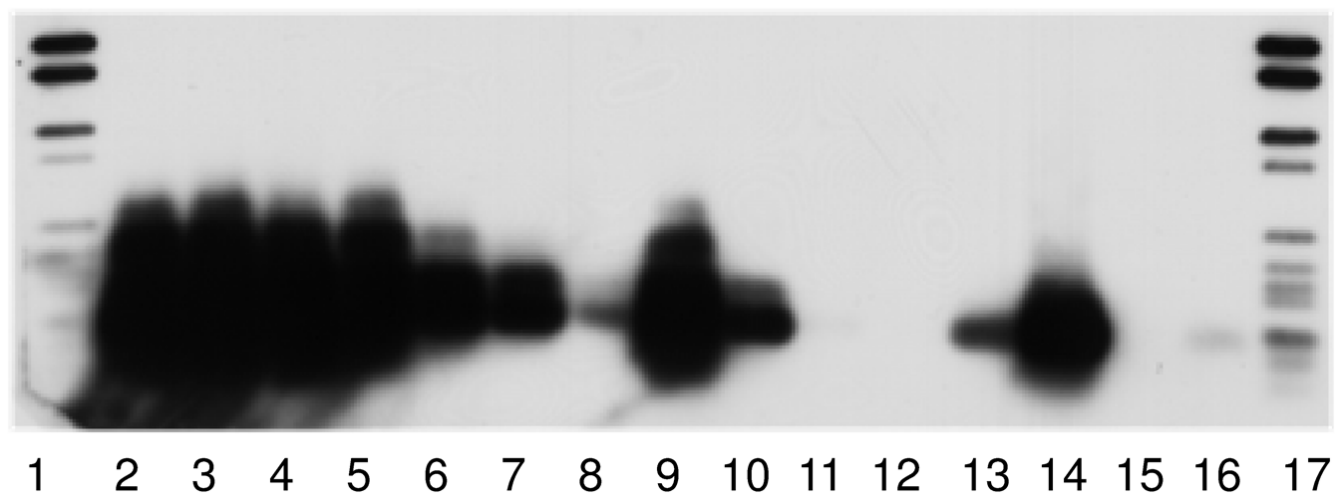
Biopurification systems (BPS). Hybridization of Southern-blotted PCR products obtained with *trfA* primer system from TC-DNA of BPS with the IncP-1ε specific probe. Lanes: 1 and 17, dig ladder; lanes 2 to 4 BPS from Lierde, Belgium; lanes 5 to 7, BPS from Kortrijk, Belgium; lanes 8 to 10, BPS from Koksijde, Belgium; lane 11, negative control; lanes 12 to 15, IncP-1 positive controls RP4 (α), R751 (β), pKJK5 (ε), pQKH54 (γ) and pEST4011 (δ). Exposure time of 5 min.

To investigate the presence of IncP-1 plasmids in different habitats a detection system based on Southern blot-PCR was applied. Using the IncP-1 mixed probe from PCR products hybridization signals of the expected size (251 bp) were detected in a very wide range of different habitats (Table 3), indicating that IncP-1 plasmids of different subgroups are widely distributed. By using probes specific for the five different IncP-1 different subgroups (α, β, γ, δ and ε), differences in the composition of IncP-1 plasmids according to the geographic area and sample type were observed. Strong hybridization signals of IncP-1α plasmids were only observed in one TC-DNA from Askö sediment (Sweden), in TC-DNA from a biogas production plant (Germany) and fosmid DNA from Baltic Sea sediments. Strong hybridization signals were observed using the IncP-1β specific probe in the TC-DNAs of all biopurification system (BPS) samples from Belgium (Table 3, Fig. 1) and most of the sediment samples from Argentina, indicating that in these environments bacterial populations carrying IncP-1β plasmids were highly abundant. The highest IncP-1γ hybridization signal was observed in the TC-DNA of the BPS located in Kortrijk. Less intense IncP-1γ hybridization signals were detected in the TC-DNAs of other BPS from Belgium and in TC-DNA of sediments from Argentina. In all TC-DNAs of BPS from Belgium, strong IncP-1δ hybridization signals were observed and a weaker hybridization signal, compared to BPS TC-DNA, was detected in TC-DNA from sediments in Argentina. Very strong IncP-1ε hybridization signals were again detected in all BPS TC-DNAs from Belgium (Table 3, Fig. 2) and most of the sediments from Argentina. Using IncP-1 mixed-probe, strong hybridization signals were detected in soils from Argentina and soil treated with chitin from the UK, indicating a high abundance of IncP-1 plasmids.

### Distribution of IncP-7 Plasmids in Different Environments

**Figure 3:**
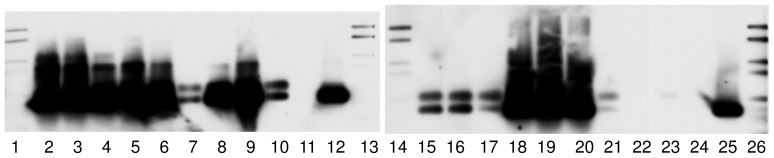
Biopurification systems (BPS). Hybridization of Southern-blotted PCR products obtained with *rep* primer system from TC-DNA of BPS with the IncP-7 probe generated from pCAR1. Lanes: 1, 13 and 26, dig ladder; lanes 2 to 4, BPS from Leefdaal, Belgium; lanes 5 to 10, BPS from Belgium (Pcfruit ); lanes 15 to 17, BPS from Lierde, Belgium; lanes 18 to 20, BPS from Kortrijk, Belgium; lanes 21 to 23, BPS from Koksijde, Belgium; lanes 11 and 24, negative control; lanes 12 and 25 IncP-7 positive control pCAR-1. Exposure time of 5 min.

To investigate the occurrence of IncP-7 plasmids in different environments, a PCR-based detection approach was applied in combination with Southern blot hybridization. Strong hybridization signals were observed in all TC-DNAs from BPS analyzed (Fig. 3), indicating a high abundance of bacterial populations carrying in BPS IncP-7 plasmid. Less intense hybridization signals were observed in the TC-DNAs of seven sediment river samples from Argentina and in the TC-DNA from soil amended with chitin from the UK. Hybridization signals using the amplicon probe specific for IncP-7 plasmids were not detected in any of the other environmental samples analyzed (Table 3).

### IncP-9 Plasmid Occurrence and Diversity in Different Environmental Samples

In order to verify the occurrence and diversity of IncP-9 plasmids in different habitats the new IncP-9 primer system developed in the present work was applied. Very strong hybridization signals were detected in all TC-DNAs of BPS samples, indicating that BPS are reservoirs of bacteria carrying IncP-9 plasmids. Less intense hybridization signals were observed in the TC-DNA of sediment samples from Argentina. A weaker hybridization signal was detected in the soil amended with chitin from the UK (Table 3).

**Figure 4:**
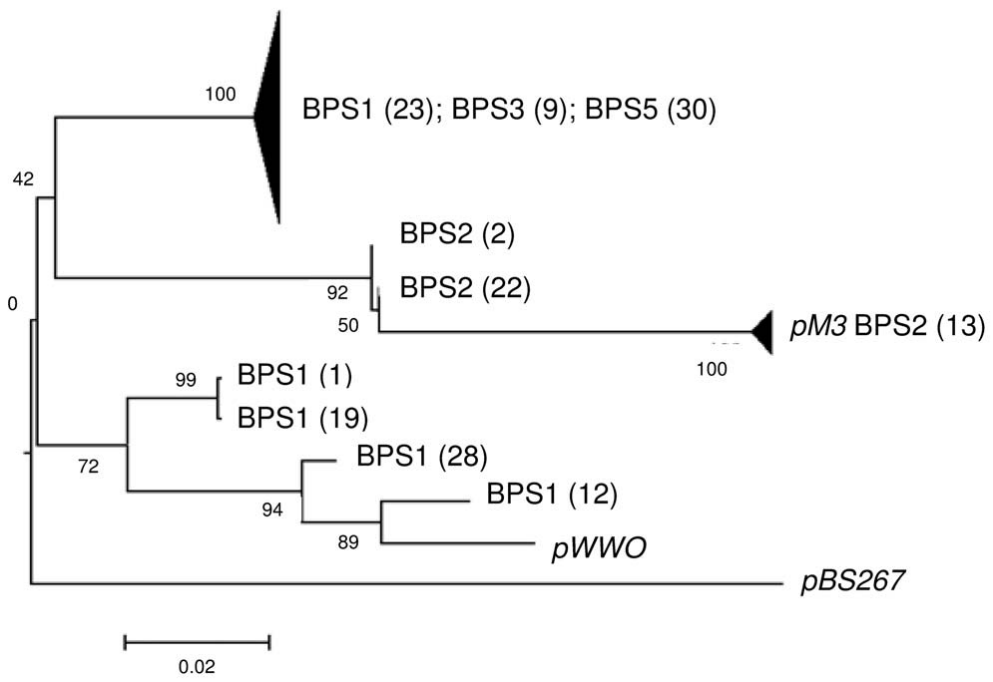
Neighbor-Joining phylogenetic tree based on the multiple alignment of cloned amplicon sequences of the *oriV-rep* IncP-9 gene. Sequences from known IncP-9 plasmids have been included as references. Value at each node is percent bootstrap support of 1,000 replicates. BPS1; BPS2 and BPS5 correspond to three different biopurification systems (BPS), located in Belgium. Numbers in brackets correspond to number of clones and numbers without brackets correspond to the clone designation.

To verify primer specificity and to gain insights into the IncP-9 plasmid diversity from BPS samples (indicated as a “hot spot” of IncP-9 plasmids), a clone library was generated with amplicons from PCR using primers targeting the IncP-9 *oriV-rep* region in TC-DNA of three different BPS. Sequencing revealed the presence of different IncP-9 subgroups while phylogenetic analysis (Fig. 4) showed IncP-9 plasmid types similar to *oriV-rep* sequences of pWWO and pM3 as well as several sequences that could not be affiliated to previously known IncP-9 plasmid groups indicating an undiscovered diversity of this plasmid group.

## Discussion

In the present study a PCR-based screening combined with Southern blot hybridization allowed the detection of IncP-1, IncP-7 and IncP-9 plasmids in a wide range of different geographic areas and sample types. The results indicated a high abundance of these plasmids in environments with different sources of pollution. It is tempting to speculate that degradative genes localized on the plasmid of these groups might contribute to the bacterial degradation of a variety of pollutants such as pesticides, due to the “metabolic complementation” resulting from the combination of different genes brought together by different plasmids. While IncP-1 plasmids typically host genes associated with the degradation of man-made pollutants (xenobiotics) [8], IncP-7 and IncP-9 plasmids often carry genes responsible for degradation of natural contaminants, such as polyaromatic hydrocarbons [9]. Screening TC-DNA revealed that IncP-1, IncP-7 and IncP-9 specific sequences vary according to sample type and degree of pollution. IncP-1 plasmid specific sequences were detected in a wide range of environments: marine sponges, soils and sediments, Baltic Sea sediment fosmid library, biogas production plant, river sediments, chitin-treated soils and BPS contaminated with pesticides. Very strong hybridization signals for all different IncP-1 subgroups tested except for IncP-1α plasmids were especially observed in the BPS samples heavily contaminated with pesticides, indicating an unusual high abundance of bacterial populations carrying IncP-1 plasmids. Indeed, the use of BPS, defined as a pollution control technique employing microorganisms to degrade pesticides through biodegradation processes [10], in on-farm treatment of water contaminated with pesticides has substantially increased and enhanced the degradation rates [11]. Strong hybridization signals of IncP-1β and IncP-1ε plasmids observed in all BPS samples and in some sediments from Argentina contaminated with oil, suggested that IncP-1β and IncP-1ε plasmids might be important in the local adaptation of bacteria to changing environmental conditions [12], [13]. Strong IncP-1 plasmid hybridization signals observed in sediments from different regions: Warwick (UK), La Plata (Argentina) and sediments from Sweden indicated that IncP-1 plasmids might also have an important ecological role in the adaptation and biodegradation processes in sediments as previously reported already for mercury-contaminated sediments in Kazachstan [14]. The apparently high abundance of IncP-1 plasmids in soils from different regions contaminated with different pollutants, such as soils from Argentina polluted with oil and soils from the UK enriched with chitin, also suggested that IncP-1 plasmids might substantially contribute to the adaptation and survival of the soil bacterial communities in response to wide range of environmental pollutants [8], [15]–[17]. The results from several studies suggested a correlation between IncP-1 plasmid abundance and pollution as hypothesized by Smalla *et al*. (2006) and confirm previously published quantitative data on the abundance of IncP-1 plasmids in BPS samples from one BPS site by means of a qPCR targeting the *korB* gene. Obviously, the relative abundance of IncP-1 plasmids can only be precisely quantified by quantitative real-time PCR. However, the recently developed *korB* quantitative PCR system [18] cannot indicate the relative abundance of the different IncP-1 subgroups which was achieved with specific probes for different IncP-1 groups used in the present study in a semi-quantitative manner.

The study by Sevastsyanovich *et al*. (2008) already showed that IncP-9 plasmid diversity is much broader than previously imagined. In view of this huge plasmid diversity, a novel IncP-9 primer system was developed and established in the present work. Typically, IncP-9 plasmids are related to the degradation of natural pollutants as polyaromatic hydrocarbons [19]. However, the detection of very strong IncP-9 hybridization signals mainly in BPS indicated that populations carrying IncP-9 plasmids are also important players in the degradation of man-made pollutants or wood-derived aromatic compounds. IncP-9 plasmids often possess different aromatic-ring degradation genes. BPS typically contain wood chips but also various aromatic ring-containing pesticides such as bentazon, epoxiconazol and diflufencian [20], which could explain the high abundance of IncP-9 plasmids observed in BPS. Cloning and sequencing of amplicons obtained with the novel IncP-9 primers from BPS TC-DNA confirmed not only the specificity of the primers but also showed the presence of plasmids with high similarity to pWWO, that were previously reported to carry degradative genes (Fig. 4) [21], [22]. The presence of several sequences with high similarity to the *oriV-rep* sequence of pM3, an antibiotic resistance plasmid belonging to the IncP-9α subgroup, in BPS 2 might be caused by manure addition in the beginning of every year (on March) by the farmers as a C-source in BPS material. Therefore, the addition of manure in BPS as nutrient source for the microorganisms might be reconsidered and replaced for an alternative one.

The indication of high abundance of IncP-9 plasmids in soils from Argentina contaminated with oil is not too surprising. IncP-9 plasmids are important vehicles for the dissemination of genes coding for enzymes involved in the degradation of polycyclic aromatic hydrocarbons (PAH) and are very often found in environments polluted with oil [23] (Flocco *et al.*, unpublished).

PCR-Southern blot hybridization results showed that bacteria hosting IncP-7 plasmids were also highly abundant in BPS, indicating a role of these plasmids in the degradation of man-made pollutants such as pesticides. It can be concluded that PCR-Southern blot hybridization detection of plasmid-specific sequences from TC-DNA is a suitable and specific but semi-quantitative approach to investigate the occurrence of plasmid-specific sequences in different environments and in a large number of samples. The detection of plasmids was possible independently of the cultivation of their original hosts [1] and indicated “hot spots” of IncP-1, IncP-7 and IncP-9 plasmids, such as BPS.

## Supporting Information

Figure S1 **Development of primer system for endpoint IncP-9 PCR of plasmid-replicon sequences.**
(TIF)
