# Supplementary figures and images for: Cultivation-Independent Screening Revealed Hot Spots of IncP-1, IncP-7 and IncP-9 Plasmid Occurrence in Different Environmental Habitats
Source: PLoS One. 2014 Feb 24;9(2):e89922. doi: 10.1371/journal.pone.0089922 (PMC3933701; doi:10.1371/journal.pone.0089922)

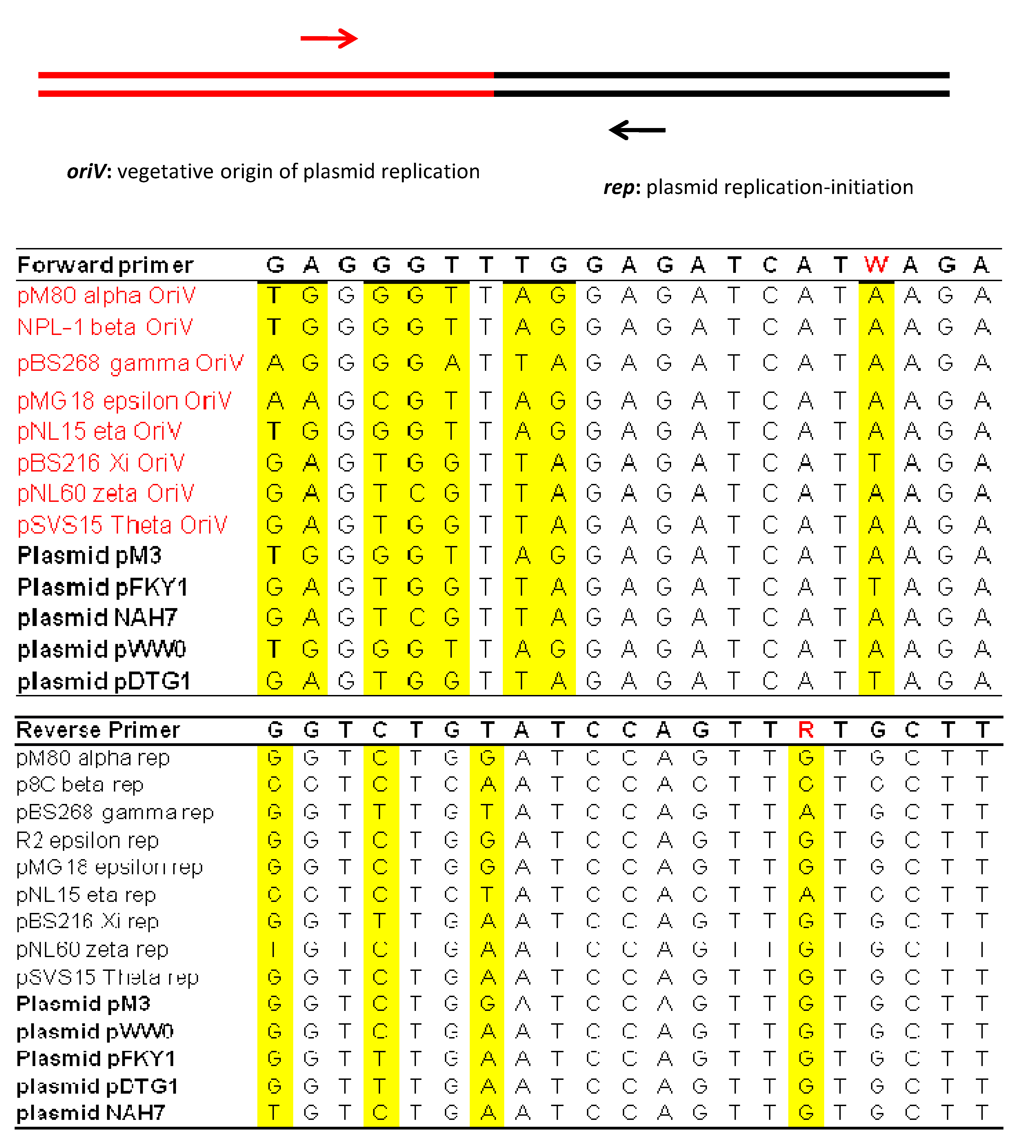

Supplement: Figure S1 — Development of primer system for endpoint IncP-9 PCR of plasmid-replicon sequences. (TIF) [file pone.0089922.s001.tif]
